# Evaluating air cleaner effectiveness in schools: a comprehensive review and protocol of a cluster-randomized controlled trial of physicochemical and microbial markers

**DOI:** 10.3389/fpubh.2026.1892902

**Published:** 2026-08-25

**Authors:** Esmée Rianne Janssen, Kimberly J. Linde, Marcel G. L. C. Loomans, Wietske Dohmen, Lützen Portengen, Lili Xia, Jan F. L. Diepens, Dick J. J. Heederik, Twan van Hooff, Inge M. Wouters, Lidwien A. M. Smit

**Affiliations:** 1 Institute for Risk Assessment Sciences, Utrecht University, Utrecht, Netherlands; 2 Department of the Built Environment, Eindhoven University of Technology, Eindhoven, Netherlands

**Keywords:** airborne microbial agents, bioaerosols, cluster randomized controlled trial (cRCT), electrostatic dust fall collectors (EDCs), indoor air quality (IAQ), particulate matter (PM), portable air cleaner (PAC), school classrooms

## Abstract

In school environments, characterized by high occupancy and prolonged exposure, airborne contaminants pose risks to respiratory health and learning outcomes. Portable air cleaners (PACs) are increasingly considered as a supplement to ventilation, yet field-based evidence remains heterogeneous, with few randomized trials and limited data on microbial agents. Here, we describe the design and methodology of a large-scale cluster-randomized controlled trial evaluating PAC effectiveness in Dutch primary school classrooms. The study included 180 classrooms across 29 primary schools, with classrooms clustered within schools and randomized to HEPA-filter PACs, ionization/plasma PACs, or no PACs. The design incorporated a three-week baseline or two-week post-intervention control period, and three repeated three-week intervention periods in the main phase, totaling up to 14 weeks per school. PACs were pre-tested under standardized laboratory conditions, screened for safety, and operated at comparable clean air delivery rates (CADR). Airborne dust was collected using electrostatic dust fall collectors (EDCs) and analyzed for bacterial markers representing common human microbiome constituents, a general bacterial indicator, and viral markers for seasonal infections. In 12 classrooms, active air sampling was conducted alongside EDCs to validate and quantify passive measurements. Continuous monitoring of particulate matter (PM₁₀, PM₄, PM₂.₅, PM₁), CO₂, air temperature, relative humidity, and volatile organic compounds (VOCs) was performed. Classroom-level absenteeism and parent-reported respiratory symptoms were collected retrospectively. Weekly national infectious-disease surveillance and outdoor PM and NO₂ data will contextualize indoor measurements and health outcomes. Hierarchical mixed-effects models accounting for school, cluster, and classroom structure will analyze microbial outcomes. Bayesian hierarchical models may be applied for values outside the quantifiable range. By integrating comprehensive indoor air quality assessment with a dual-control design distinguishing pre-existing classroom differences from temporal trends, this registered trial (ClinicalTrials.gov, NCT07479420; 6 March 2026) provides a rigorous framework to evaluate PACs under real-world classroom conditions and support evidence-informed strategies to improve classroom indoor air quality.

**Clinical trial registration**: https://clinicaltrials.gov/study/NCT07479420?term=air%20cleaner&viewType=Card&intr=air%20cleaner&rank=4, ClinicalTrials.gov, NCT07479420.

## 1 Introduction

Airborne contaminants, including infectious agents such as viruses and bacteria, as well as non-infectious pollutants like particulate matter (PM) and chemicals, pose a critical public health risk in indoor environments, especially in densely occupied, poorly ventilated spaces with prolonged exposure times, such as schools (1). Poor indoor air quality (IAQ) may exacerbate respiratory symptoms, asthma, and reduce cognitive performance among students (2–5). The COVID-19 pandemic highlighted the role of schools in community outbreaks (6, 7) and demonstrated the high costs of prolonged closures, including learning loss, increased educational inequality, and mental health impacts (8–11). Consequently, improving IAQ is critical for safeguarding health, enhancing educational outcomes, and mitigating the impacts of both routine and outbreak-related exposures in schools.

In the Netherlands, during the last decade school IAQ has received increased regulatory attention, with mandatory CO₂ monitoring by July 2025, a minimum ventilation rate of 6 dm^3^/s per person (12, 13), and national guidelines recommending CO₂ concentrations below 1,200 ppm during occupancy (14, 15). Nonetheless, classroom air quality often falls short. In a 2020 study of 31 classrooms in 11 Dutch secondary schools, 42% of all classrooms exceeded the Dutch national CO₂ guidelines during occupancy, and 13% had a ventilation rate below the required 6 dm^3^/s per person (16). In the absence of recent PM data for Dutch classrooms, the most recent evidence comes from a 2010–2012 study of 18 classrooms across 17 primary schools, where weekly average PM₂.₅ and PM₁₀ concentrations were 17.4 μg/m^3^ and 52.1 μg/m^3^, respectively (17). These levels exceed current WHO health-based guideline values (annual average limits of 5 and 15 μg/m^3^ for PM₂.₅ and PM₁₀, and 24-h limits of 15 and 45 μg/m^3^, respectively) (15, 18). Although these guidelines apply to ambient air, exceedances indoors still highlight potential air quality concerns.

While regulatory standards in the Netherlands have primarily focused on physical and chemical indicators of indoor air quality, microbial thresholds are lacking and quantitative data on classroom airborne microbial concentrations remain limited. Previous studies have primarily reported endotoxin (4) and total bacterial concentrations (19–21). Nonetheless, the COVID-19 pandemic emphasized the importance of recognizing airborne virus transmission in school classrooms, including the spatiotemporal dynamics of infectious respiratory particle (IRP) behavior and indoor dispersion that influence exposure risk (22). IRPs span a range of sizes, with smaller particles remaining suspended longer and dispersing more widely indoors (23, 24). These particle dynamics, combined with indoor airflow patterns and room geometry create pronounced spatial heterogeneity that influences exposure risk and the performance of portable (i.e., stand-alone, non–system-integrated) air cleaners (PACs) (25), which can attenuate room-average concentrations but are less effective at preventing short-range exposure.

While ventilation remains essential to improve IAQ, PACs may contribute as a complementary, cost-effective (26) and rapidly deployable measure, offering an additional layer of protection to help mitigate endemic respiratory infections and provide temporary support during seasonal outbreaks. In line with the American Society of Heating, Refrigerating and Air-Conditioning Engineers guidelines, PACs can augment existing ventilation strategies by contributing to the total equivalent clean air delivery, enhancing IAQ and reducing airborne exposure risk (27). PACs employ different airflow configurations and filtration technologies, including: (1) high-efficiency particulate air (HEPA) filtration, which mechanically traps particles through dense fibrous filters (28); (2) ionization systems, including electrostatic precipitators, which charge particles to promote agglomeration or surface deposition (29, 30); (3) plasma-based systems, generating reactive species that inactivate microorganisms or cluster particles onto surfaces (31); and (4) ultraviolet-based systems, including UV-C radiation and photocatalytic oxidation, which neutralize microorganisms with ultraviolet light (32) or degrade biological contaminants via UV-activated catalysts (33).

Chamber-based laboratory studies demonstrate that PACs can reduce airborne viral particles, including aerosolized SARS-CoV-2, under controlled conditions (34, 35). In simulated classrooms, containing typical furniture, aerosolized phage concentrations decreased by ~90% within 30 min; however, also without PACs, substantial removal occurred via ventilation or deposition (36), suggesting that the incremental benefit of PACs was modest. Moreover, these laboratory results reflect idealized conditions that rarely occur in occupied classrooms. Real-world airflow patterns, furniture layout, human activities, and ventilation heterogeneity influence aerosol dispersion and deposition, meaning that laboratory-derived efficiencies likely represent the upper bound of achievable performance, underscoring the need for field-based validation (36).

Consistent with laboratory findings, real-world studies in schools confirm that PACs can reduce overall aerosol concentrations, which encompass the full range of airborne particle sizes and types present in classrooms (Table 1; see Supplementary Table S1 for search strategy). In occupied classrooms, naturally occurring aerosol concentrations decreased rapidly across a broad range of particle sizes, with reductions of approximately 84 to 95% within 20–30 min (37–39). Similarly, a crossover study reported an average 77% reduction in aerosols when comparing PAC-on versus PAC-off periods (38). However, some of these reductions were observed under closed-window conditions or without accounting for natural decay, and in some cases, initial aerosol concentrations were moderate, meaning large relative reductions can correspond to smaller absolute changes.

**Table 1:** Characteristics and outcomes of published air cleaner studies in schools.

Reference	Year	Location	Study sites	Intervention	CADR (m^3^/h)	Study design	Duration^6^	Airborne agent	Outcome
Overall aerosol concentrations (all measured particle sizes)									
Curtius et al. (37)	2021	Germany	2 classrooms, 1 secondary school	HEPA PACs (4/classroom)	973 ± 150^1^	Between-group classroom comparison (during closed-window period)	5 d	Particle mass, particle number (>3 nm, 10 nm–10 μm)	↓ particle mass 84% (37 min); ↓ particle number >3 nm > 90% (30 min)
Banholzer et al. (38)	2024	Solothurn, Switzerland	2 classrooms, 1 secondary school	HEPA PACs (2/classroom)	420/device ^1^	Crossover (classroom-level)	7 wks (alternating interventions)	Particle number (175 nm–20 μm)	↓ particle number 77% (Bayesian 95% CrI 63–86%)
Granzin et al. (39)	2022	Bad Homburg, Germany	2 classrooms, 2 schools (primary/secondary)	HEPA PACs	610–1,050 ^2^	Natural decay measurements directly after window closure	6 mo	Particle number (0.003–10 μm)	↓ particle load 85–95% within ~20 min between ventilation periods
PM mass									
Jehn et al. (40)	2024	Arizona & Connecticut, USA	53 classrooms, 5 schools (primary/secondary)	DIY PACs	170–600 (mean 406) ^1^	Repeated-measures aerosol decay experiments (ON vs. OFF)	Multiple decay experiments over 6 mo	PM₂.₅, PM₁₀	↓ PM₂.₅ 43.3%,↓ PM₁₀ 30.6%
Simona et al. (41)	2025	Los Angeles, USA	186 classrooms, 17 primary schools	HEPA PACs	NR ^4^	Block randomized crossover (classroom-level)	2 yr. (condition crossover after 1 yr)	PM₁, PM₂.₅, PM₁₀	Schoolyear avg.: PM₁ NS; ↓ PM₂.₅ 39.9%; PM₁₀ NS
Azevedo et al. (42)	2022	Rhode Island, USA	2 classrooms, 1 primary school	HEPA PACs + A/C fans	Min: NR Max: 950 ^2^	Half-day randomized PAC at high vs. low (classroom-level)	11 school d	PM₁, PM₂.₅	PAC high vs. low: ↓ PM₁ 0.38 μg/m^3^; ↓ PM₂.₅ 0.10 μg/m^3^
Han et al. (43)	2022	Seoul, Korea	12 classrooms, 2 primary schools	8 PAC types (1-2/classroom)	594–1,278 (mean 909) ^1^	Clustered classroom comparison (3 classrooms / cluster: 0, 1, 2 PACs; repeated per PAC model)	All classrooms measured simultaneously; each PAC model tested twice	PM₂.₅, PM₁₀	1 PAC: PM₂.₅ ↓ 67.0%, PM₁₀ ↓ 58.3%; 2 PACs: PM₂.₅ ↓ 81.0%, PM₁₀ ↓ 77.5%
Choe et al. (104)	2022	Daegu, Korea	32 classrooms 24 schools (primary/middle/high)	HEPA PACs	600–900 (flow rate; CADR NR) ^3^	Pre-post (classroom-level)	6 mo	PM₂.₅, PM₁₀	↓ PM₂.₅ 25–33%; ↓ PM₁₀ 23–31%
Dong et al. (44)	2019	Beijing, China	6 classrooms, 1 middle school	Ionization PACs	NR ^4^	Crossover (classroom-level, double-blind randomized)	5 d PAC, 5 d SHAM ^7^, 2 mo washout	PM₂.₅, PM₁₀	↓ PM₂.₅ 44%; ↓ PM₁₀ 34% vs. SHAM ^7^
Xia et al. (45)	2024	Detroit, USA	21 classrooms, 2 schools (primary/middle)	HEPA PACs	255–1,087 ^1^	Pre-post (classroom-level)	1 wk. baseline, 1 wk. follow-up, 4–5 wk. PAC	PM₂.₅, PM₁₀	↓ PM₂.₅ 28–76%; ↓ PM₁₀ 28–76%
Phipatanakul et al. (46)	2021	North-eastern USA	209 classrooms, 41 primary schools	HEPA PACs (4/classroom)	180/device ^1^	cRCT with baseline sampling ^5^	1 school yr.: 3 × 1 wk. sampling	PM₂.₅, coarse PM (PM₂.₅–PM₁₀)	↓ PM₂.₅ 1.7 μg/m^3^ (95% CI –2.5 to −0.9); ↓ coarse PM 2.2 μg/m^3^ (95% CI –3.0 to −1.4)
Villanueva et al. (47)	2024	Ciudad Real área, Spain	19 classrooms, 7 schools (range pre-secondary)	HEPA PACs	270–330 ^2^	Pre–post (classroom level)	3 mo	PM₂.₅, PM₁₀	No consistent reduction
Park et al. (48)	2020	Korea	102 classrooms, 34 primary schools	PAC (type unspecified)	NR ^4^	Between-group classroom comparison (observational, non-randomized)	19 schools: 2 mo, 15 schools: 1 mo	PM₂.₅, PM₁₀	↓ PM₂.₅ 36%; ↓ PM₁₀ 34%
Rawat & Kumar (49)	2024	Guildford, UK	2 classrooms, 1 infant school	HEPA PACs	119 ^3^	Pre–post (classroom-level)	Baseline 5 d Intervention 2 d	PM₁, PM₂.₅, PM₁₀	↓ PM₂.₅ 17%, ↓ PM₁₀ 18% (classroom B); no significant reduction in classroom A
Abhijith et al. (50)	2022	London, UK	3 primary schools (1 classroom with PACs)	HEPA PACs (2/classroom)	NR ^4^	Pre–post (classroom-level)	Baseline 2 wks 2 wks intervention	PM₁, PM₂.₅, PM₁₀	↓ PM₁ 19–23%; ↓ PM₂.₅ 23–48%; PM₁₀ 49–57%
Overall aerosol concentrations (all measured particle sizes) and microbial agents									
Basińska et al. (54)	2021	Swarzędz, Poland	2 classrooms, 1 primary school	Ionization PACs	100–250 ^2^	Pre–post (classroom-level, daily pre-PAC ON vs. post-PAC ON)	2 mo	Particle number (0.3–10 μm), bacteria, fungi	No consistent reduction
Brągoszewska & Biedroń (53)	2021	Gliwice, Poland	1 kindergarten	HEPA PACs	310 ^3^	Repeated within classroom comparison (ON vs. OFF)	6 mo (repeated PAC off/ on periods)	Bacteria, respirable fraction <3.3 μm	↓ bacteria18%; ↓ respirable fraction 20%
PM mass & microbial agents									
Juntunen et al. (51)	2025	Kuopio, Finland	27 classrooms, 6 primary schools	HEPA or EPA PACs	130–331^3^	Crossover (classroom-level, randomized	6 wk. per classroom: 2 wk. SHAM ^7^/2 wk. OFF / 2 wk. ON	PM₂.₅, PM₁₀, bacteria, fungi	↓ PM₂.₅ 24.8%; PM₁₀ NS; bacteria NS; fungi NS
Oh et al. (52)	2014	Seoul, Korea	20 classrooms, 10 childcare centers	HEPA PACs	NR ^4^	Pre-post (classroom level)	5 d baseline, 3 wk. PAC per classroom	PM₂.₅, PM₁₀, bacteria, fungi	↓ PM₂.₅ 86%; ↓ PM₁₀ 69%; ↓ airborne bacteria 62%; ↓ airborne fungi 55%
Sun et al. (55)	2025	North-eastern USA	200 classrooms, 39 primary schools	HEPA PACs (4/classroom)	NR ^4^	cRCT with baseline sampling ^5^	1 school yr.: 3 × 1 wk. sampling	Respiratory viruses (19 targets)	No reduction in viral exposure (OR 0.50; 95% CI 0.08–3.25); ↓ viral diversity 32.8%

1 Study-estimated CADR values.

2 Manufacturer-reported CADR values.

3 Not specified how CADR is determined.

4 NR, not reported.

5 cRCT, cluster-randomized controlled trial.

6 D, days; wks, weeks; mo, months; yr., years.

7 SHAM, control condition mimicking; PAC, presence without effective air filtration.

Additionally, numerous in-situ studies examined changes in PM concentrations during normal occupancy (40–52) (Table 1). Across most studies, PAC operation was associated with reductions in PM₁, PM₂.₅ and PM₁₀, although effect sizes varied substantially. Larger and more consistent reductions were generally reported in studies using higher clean air delivery rates (CADRs; the volume of filtered air delivered per unit time (m^3^/h), reflecting both filter efficiency and airflow rate) or multiple PACs per classroom, although CADR was not consistently reported in all studies. A small number of studies (47, 49) observed no consistent reduction or effects limited to a subset of classrooms, potentially reflecting classroom-specific conditions, PAC configurations and CADR, or study design. Despite this heterogeneity, the collective evidence indicates that PACs can substantially reduce PM concentrations in real-world school environments.

Literature on in-situ studies examining PAC effects on microbial agents in schools is more limited, and reported effects vary markedly across studies and settings (Table 1). Reductions in airborne bacteria and fungi have been reported in some studies, with decreases ranging from 18 to 62% for bacteria and 55% for fungi (52, 53), both employing active agar-based sampling methods. In contrast, studies using quantitative polymerase chain reaction (qPCR) analysis of settled dust or active filter-based air sampling report minimal effects on bacterial, fungal, or viral concentrations (51), though a decrease in viral diversity was suggested (54, 55). Overall, the available evidence suggests that PACs may reduce airborne microbial concentrations under real-world classroom conditions, but effects are less consistent than those observed for particulate matter, highlighting the need for further targeted research.

While several large studies have examined PM outcomes, randomized controlled trials remain scarce, and evidence on airborne microbial responses is inconsistent. To address these gaps, a large-scale cluster-randomized controlled trial (cRCT) is conducted in Dutch primary schools, combining pre-intervention measurements, control classrooms, and extended monitoring of airborne microbial communities, PM and multiple other physicochemical parameters. The study incorporates bacterial markers representing common components of the human-associated microbiome, a general indicator of total bacterial load, and selected respiratory viruses relevant to seasonal infection dynamics. This article outlines the study design, recruitment process, descriptive characteristics of the participating schools and classrooms, and the methods, including PAC selection and planned data analyses, providing a framework for one of the most comprehensive real-world assessments of PAC effectiveness in educational settings to date.

## 2 Study design and setting

**Figure 1:**
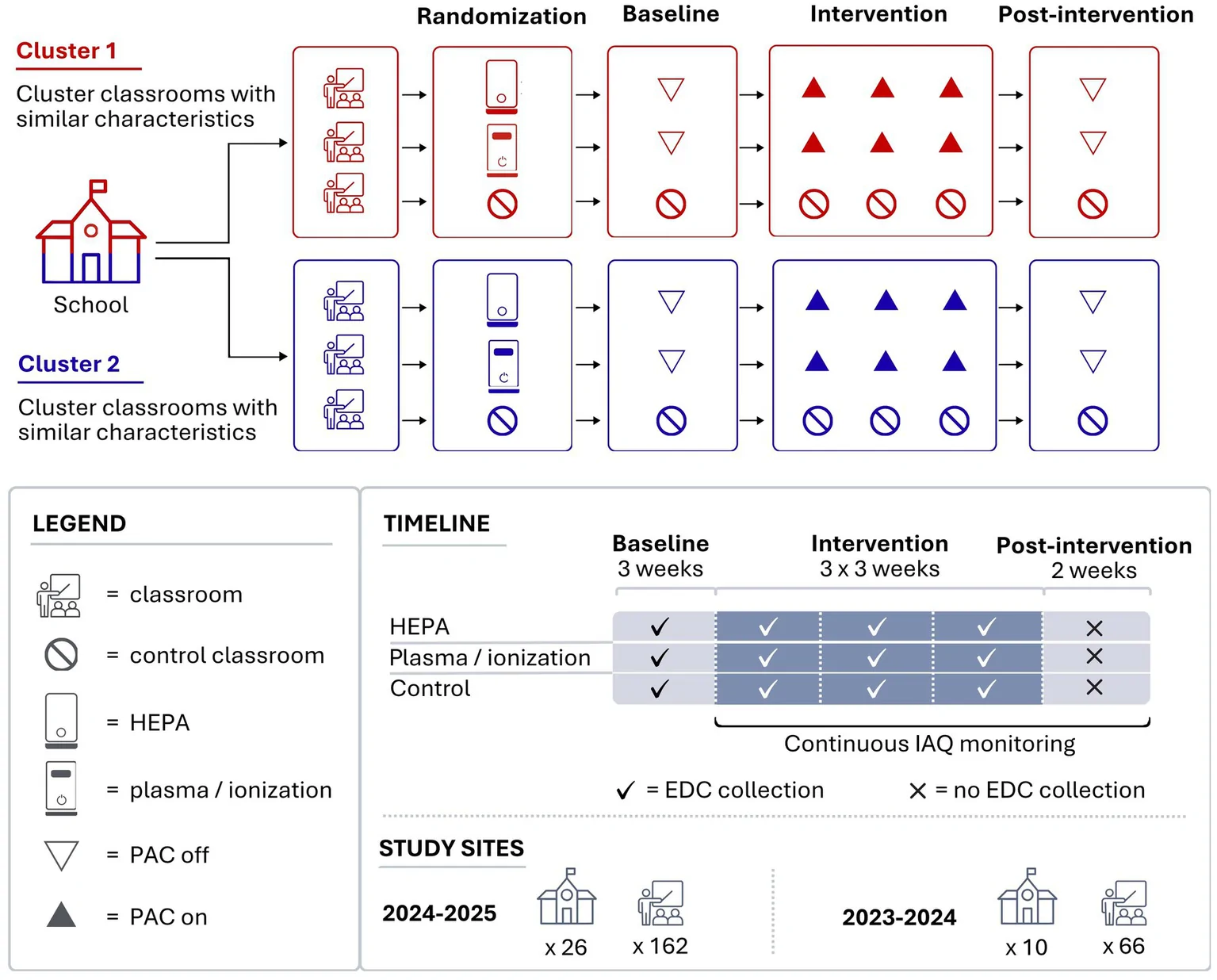
Study design and timeline of the intervention cluster-randomized controlled trial. Classrooms with similar characteristics were grouped into clusters and randomly assigned one of three study conditions: HEPA filter, ionization/plasma, or control. The 14-week study included a 3-week baseline (PACs off), three 3-week intervention phases (PACs on), and a 2-week post-intervention period (PACs off).

A cRCT was conducted with randomization within schools at the classroom level to minimize confounding from school-level factors. The study comprised two phases: an initial phase (2023–2024) including 10 schools (66 classrooms), and a main study period (2024–2025) including 26 schools (162 classrooms). As shown in Figure 1, classrooms within a school were manually clustered to optimize comparability, with each school contributing one or more clusters and each cluster restricted to classrooms within the same building and sharing identical ventilation systems and flooring types. Flooring types were matched specifically to avoid differences in particle resuspension and aerosol dispersion. Following these primary clustering criteria, additional classroom characteristics, including floor level, orientation, classroom size, student number and age group, were considered.

Each cluster was then randomly assigned to one of three study conditions: (1) HEPA filter PACs, (2) ionization/plasma PACs, or (3) control, i.e., no PACs (Figure 1). A dual-control design was used, with a control sample collected in each classroom during either the three-week baseline period prior to the intervention or the two-week post-intervention period, alongside a continuously monitored control group throughout the full 14-week study (Figure 1). In the initial study phase, the number and duration of intervention periods varied by school (ranging from one to four interventions), whereas in the main study period, each classroom underwent a three-week baseline followed by three repeated three-week intervention periods.

### 2.1 School recruitment and selection

**Figure 2:**
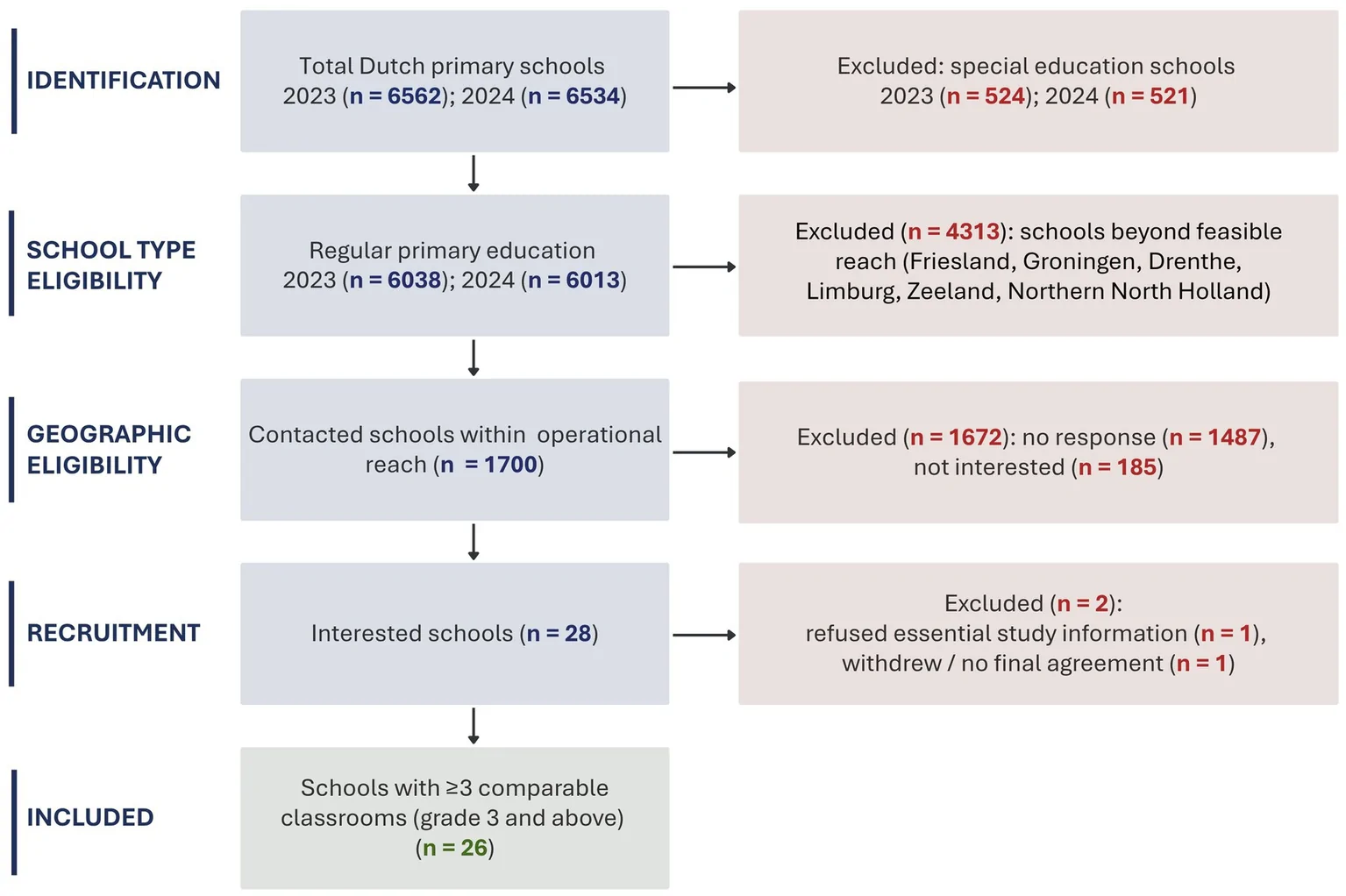
Flowchart showing the recruitment and selection of schools for inclusion in the cRCT. Schools were included if they had at least three comparable classrooms; Kindergarten classes were excluded due to differences in occupancy patterns and activity types affecting bioaerosol dynamics (57).

For the main study period (2024–2025), out of an estimated 6,500 primary schools in the Netherlands (56), approximately 1,700 primary schools within feasible operational reach of Utrecht were contacted. Schools for special education and Kindergarten classes (children aged ~4–6 years) were excluded. Of the contacted schools, 213 replied; 185 declined participation, and 28 expressed interest. Schools were included if they had at least three comparable classrooms to be randomized in the study. Two schools were later excluded due to process-based reasons: one school refused to provide essential study information, and one withdrew before final agreement. The final study population of the main study period comprised of 26 schools. The inclusion/exclusion process is depicted in Figure 2.

**Figure 3:**
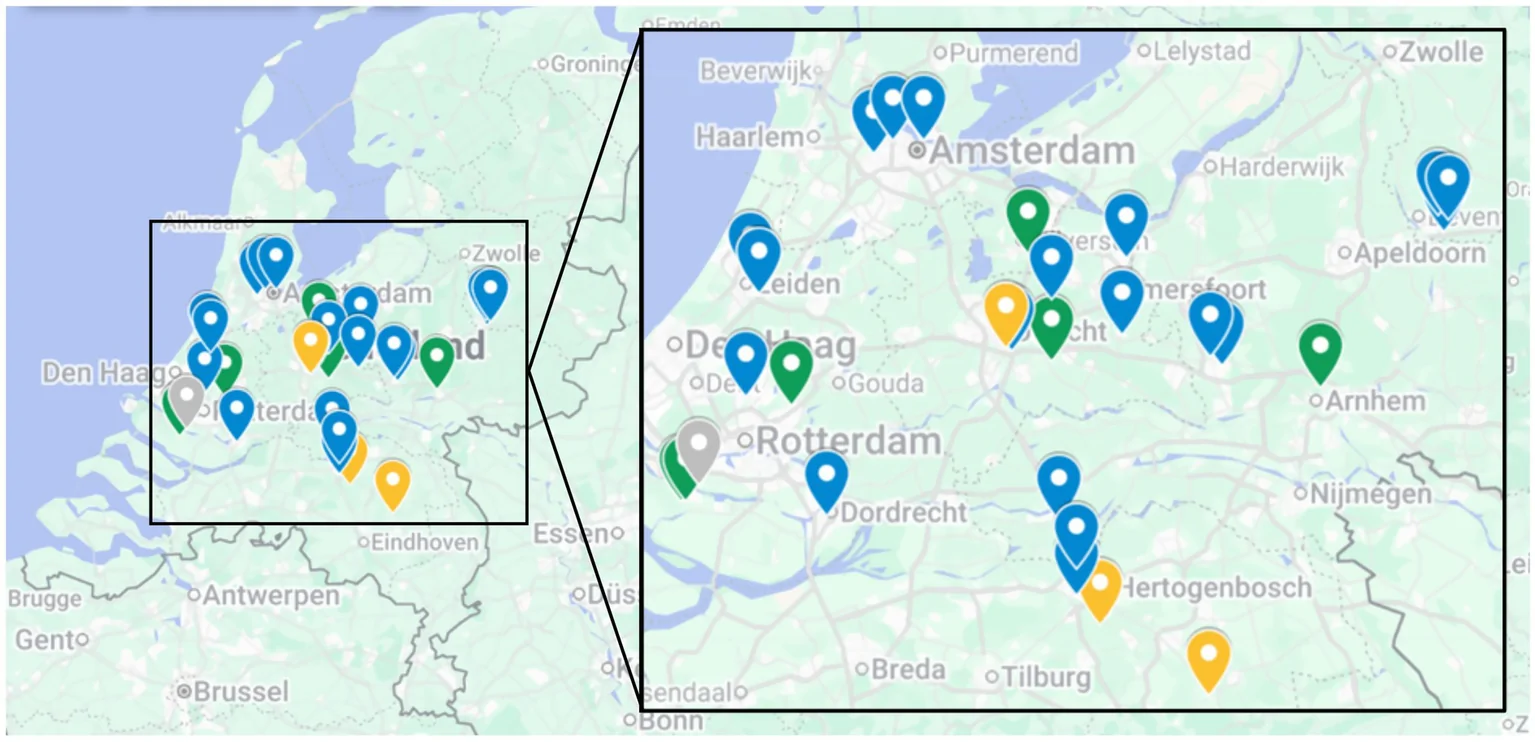
Geographic distribution of participating and excluded schools across study periods. Orange pins indicate the initial-phase sites (2023-2024), blue the main-phase sites (2024-2025), green those participating in both phases, and a gray pin denoting a school excluded due to non-comparable measurement timing.

Participating schools were spread across multiple provinces in the Netherlands (Figure 3) and varied in classroom sizes, ventilation systems, student numbers, and special features such as flexible grouping and air conditioning units (Table 2). Ventilation type was classified as natural, mechanical (balanced with supply and exhaust), mechanical (exhaust only), or unknown, based on interviews with school contacts and on-site observations. An initial study phase (Dec 2023-May 2024) was conducted in 11 schools and 69 classrooms; one school from this phase is to be excluded from subsequent analyses due to a prolonged delay between baseline and intervention measurements, rendering them temporally non-comparable with the other schools due to seasonal differences in viral circulation and classroom ventilation behavior. This results in 10 schools and 66 classrooms in the initial phase. The main study period (Oct 2024–May 2025) comprised 26 schools and 162 classrooms. Across both study phases, 180 classrooms participated, with 48 of the classrooms participating in both years.

**Table 2:** Study characteristics of participating schools.

Characteristics	Initial study phase (2023–2024)	Main study phase (2024–2025)
Schools (n)	10	26
Geographic setting ^1^		
Extremely urbanized	10.0%	26.9%
Strongly urbanized	30.0%	23.1%
Moderately urbanized	20.0%	30.8%
Hardly urbanized	40.0%	19.2%
Classrooms (n)	66	162
Classroom surface area (m^2^) ^2^	50.3 [47.0–56.0], 38.9–60.6	51.4 [42.4–56.1], 35.1–76.4
Classroom volume (m^3^) ^2^	157.5 [149.4–186.6], 115.0–236.7	156.2 [129.2–182.0], 95.1–242.9
Ventilation system		
Natural	22.7%	11.1%
Mechanical (balanced)	22.7%	3.7%
Mechanical (type unknown)	27.3%	74.1%
Mechanical (exhaust only)	27.3%	11.1%
Special features		
Carpet flooring	2 clusters	2 clusters
Open-plan learning spaces	N. A.	1 school
Combination class in afternoons	N. A.	1 school
Air conditioning units	2 classrooms	2 classrooms
Students per classroom ^2^^,^ ^3^	27 [23–28], 17–32	24 [22–27], 9–37
Grade ^2^	6 [5–7], 3–8	6 [4–7], 3–8

1 Urbanization level classified according to address density (addresses per km^2^) as defined by Statistics Netherlands (CBS): extremely urbanized (≥ 2,500), strongly urbanized (1,500–2,500), moderately urbanized (1,000–1,500), hardly urbanized (500–1,000), and non-urbanized (rural) (< 500) (105).

2 The following is reported: median [IQR], min–max.

3 Student numbers per classroom were determined via desk counts and could fluctuate across measurement periods; values were averaged per class, with min and max reflecting the lowest and highest observed counts in any period.

## 3 Air cleaner selection, deployment and monitoring

### 3.1 Air cleaner selection

**Figure 4:**
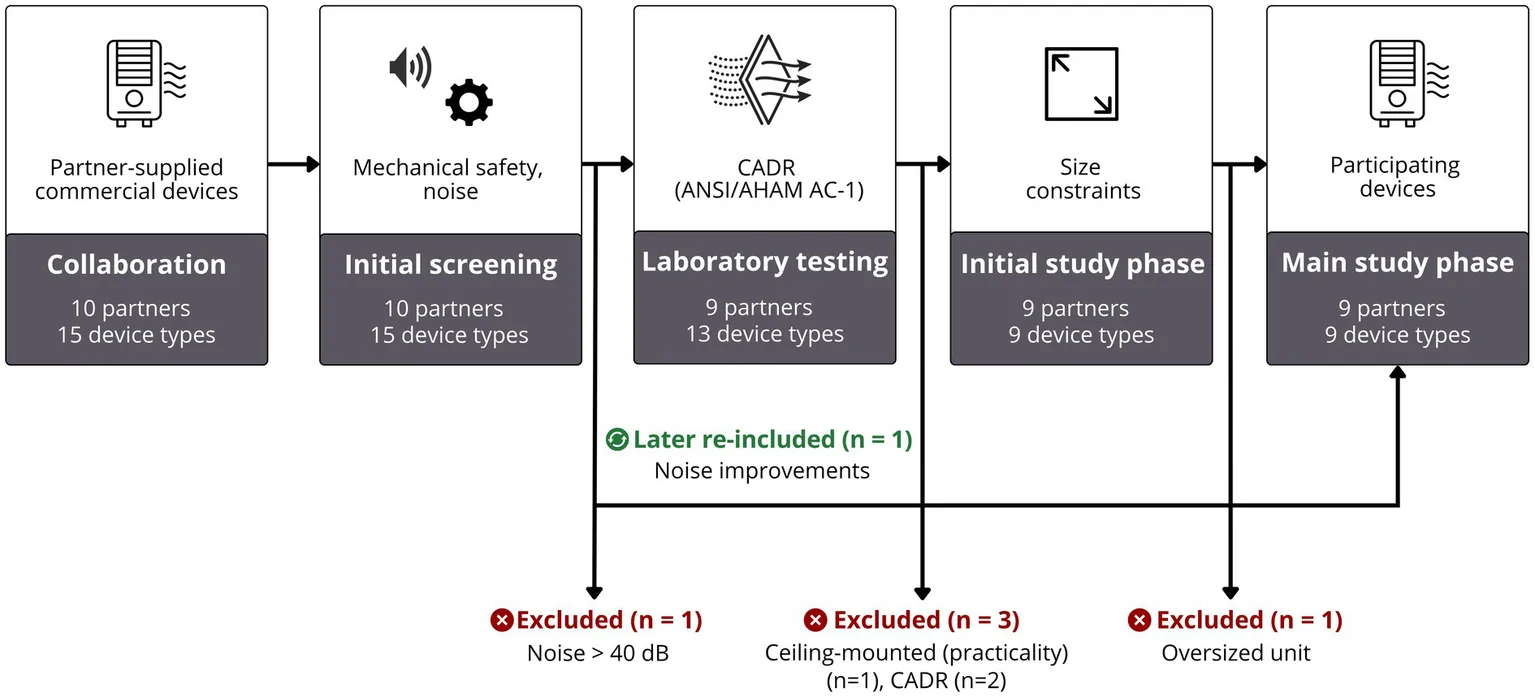
Stepwise screening and selection of mechanical air cleaners (PACs) for the randomized controlled trial. Devices were evaluated for mechanical safety, noise, CADR performance, and size constraints. Non-ceiling-mounted devices were favored for practical reasons; one ceiling-mounted device type was included as it constituted the exclusive device type supplied by this partner company.

PACs were selected from commercially available devices provided by partner companies who participated in this public-private partnership that aimed to expedite the development of advanced air-cleaning technologies and provide empirically grounded feedback to manufacturers on device performance. Partner companies were not involved in execution of the research allocation procedures nor the delineation of the results. Their involvement was restricted to providing installing and maintaining the systems at the selected schools executed in a manner consistent with their normal client operations. Each partner company was aware only of systems installed in its assigned schools and had no knowledge of installations by other partners in the remaining schools. Randomization of PAC types to schools was implemented via an algorithm that weighted selection based on device availability from each partner while ensuring that all partner companies were represented at least once. Allocation was finalized before it was disclosed to participating schools and partner companies. In 29 out of 180 classrooms practical constraints such as device size or noise were considered to accommodate specific classroom preferences expressed by the school. Prior to formal laboratory testing all PACs were evaluated for basic mechanic integrity and classroom feasibility considering manufacturer-reported noise levels typically below 40 dB (Figure 4).

PACs included in the cRCT employed different airflow configurations and filtration technologies. All selected devices underwent formal pre-testing in highly controlled laboratory settings at Eindhoven University of Technology (TU/e) with CADR as the primary performance metric following the ANSI/AHAM AC-1 standard procedure (57). Tests were conducted in a standardized 28.4 m^3^ chamber where each device was tested in triplicate under well-mixed conditions. The testing protocol is described in detail elsewhere (58). Subsequent inclusion in the cRCT was limited to devices demonstrating adequate CADR performance noise levels and safety standards. Settings of all devices were adjusted in the cRCT to achieve a comparable CADR of 400–500 m^3^/h which corresponds with approximately a 20% improvement over the required ventilation flow rate performance according to Class B as described in the Dutch program of requirements (PvE) for good indoor environmental quality in schools (15). For one type of PAC two devices were needed to reach the required 400–500 m^3^/h

In addition to performance testing, device safety was a critical selection criterion. Previous work has raised concerns about potential byproducts from some air cleaning technologies. Ionization (59–62) and plasma devices (63–66) may generate ozone, which may react with common indoor volatile organic compounds (VOCs) to form secondary byproducts such as formaldehyde and ultrafine particles (59), potentially posing respiratory health risks. Based on these considerations, prior to deployment all units underwent supplementary ozone testing using an Aeroqual device equipped with ozone sensor. Measured ozone concentrations were below detection limits, suggesting formation of secondary reactive species via ozone-mediated pathways is likely minimal.

During the initial study phase (Dec 2023–May 2024), some oversized units were trialed, but based on feedback from schools regarding classroom practicality, these were excluded from the larger cRCT.

### 3.2 Air cleaner deployment and monitoring

PACs were intended to be positioned according to the partner companies’ recommendations for optimal cleaning efficiency which varied between devices due to differences in airflow configuration and design. However in practice placement was largely dictated by practical constraints such as available space classroom layout and to a minor extent by availability of power outlets. Consequently PAC placement was not fully standardized across classrooms but rather reflected realistic implementation conditions in occupied school environments. Teachers were instructed to keep the devices operational at all times and asked to document any temporary shutdowns or adjustments to lower settings on a standardized log form. Beyond this schools could continue their usual routines and no modifications to ventilation practices were instructed. In addition during each three-week routine sample collection (described below) the operational status and settings of the devices were systematically recorded by the research team. For schools using the PACs over extended periods devices requiring maintenance were attended to after the summer holiday following the manufacturer’s standard procedures to ensure consistent performance. Additionally TU/e conducted follow-up performance testing of all devices at the end of the study period to verify sustained air cleaning efficacy. Blinding of students and school staff was not feasible as air cleaners without active filtration could not be provided across all participating partner companies.

## 4 Sample collection and measurements

### 4.1 Airborne dust sampling

Airborne dust was passively collected using electrostatic dust fall collectors (EDCs) following the standardized protocol by Noss et al. (67). Each EDC consisted of two electrostatic cloths (purchased from Albert Heijn, The Netherlands) which were sterilized by heating at 220 °C for 4 h enclosed in an ethanol-cleaned polypropylene folder. Prior to deployment, EDCs were packaged in closed bags. EDCs were typically suspended from the classroom ceiling (> 0.3 m below ceiling, ≥ 1.80 m above floor surface), positioned centrally and away from windows, doors, PACs, lighting fixtures, and ventilation inlets/outlets. In a subset of classrooms (19 of 180), placement was adapted due to practical constraints. In most of these cases (15 of 19), the adapted placement was approximately standardized within classroom clusters, such that all classrooms within a cluster used similar EDC positioning. Samplers were exposed for three-week intervals over one baseline and three consecutive repeated intervention measurements, resulting in a total sampling period of 12 weeks for the EDCs (Figure 1). To monitor background contamination, sealed field blanks (32 initial phase, 113 main study) and laboratory blanks (38 initial phase, 77 main study) were included.

### 4.2 Laboratory analysis and microbial targets

Following sample collection, EDC cloths were removed from the holders individually packed in bags and stored at −80 °C until Deoxyribonucleic acid (DNA) and ribonucleic acid (RNA) extraction. Samples were thawed, mixed with 10 mL of 1:1 LGC lysis buffer BL (LGC Genomics GmbH) and Phosphate Buffered Saline (PBS; Gibco™ 1 × Dulbecco’s phosphate-buffered saline without calcium, magnesium, and phenol red; Fisher Scientific), agitated on a roller for 30 min, and placed on ice. Bacterial and viral targets were extracted from 350 μL of supernatant using commercially available kits (see Supplementary Table S2 for detailed reagent information): the LGC DNA kit [LGC Genomics GmbH; per Fakunle et al. (68)] was used to extract total nucleic acids (DNA and RNA), while the Zymo Quick-RNA MiniPrep Kit (Zymo Research, supplied by Sanbio) was used to extract RNA with DNA removal, according to supplier instructions.

Bacterial and viral targets were analyzed in technical duplicates, respectively, by qPCR and reverse transcript qPCR on a BioRad CFX 384 (initial phase) or QuantStudio (main phase) machine with negative controls and 50 PCR cycles (see Supplementary Table S3 for detailed assay conditions). Standard curves of synthesized gBlock fragments were included to enable quantification. Quality-control checks will report blank contamination (counts, percent detectable, and mean blank levels) and duplicate agreement (mean difference, limits of agreement, correlation metrics); handling of any background contamination will be described. Limits of detection (LOD) and quantification (LOQ) will be reported. Laboratory personnel were blinded to intervention allocation.

Bacterial targets (*Staphylococcus aureus*, *Streptococcus salivarius*, *Staphylococcus epidermidis*, and *Moraxella catarrhalis)* represent common constituents of the human microbiome across the respiratory tract, mouth and skin. Viral targets (respiratory syncytial virus (RSV), Influenza A and B) were selected as representative agents of prevalent seasonal respiratory infections. Although the study was initially conceptualized in the context of COVID-19, SARS-CoV-2 was not included due to challenges in consistent airborne detection at the time of the field study, and to broaden the scope toward airborne microbial exposure and infection prevention more generally.

### 4.3 Active air sampling sub-study

To complement passive dust collection, active air sampling was conducted in a sub-study to support quantification and validation. In a subset of 12 classrooms at one school, active air sampling was performed using Derenda pumps equipped with Harvard impactors and Teflon filters (Pall 2.0 um pore-size), operated continuously at 0.20 m^3^/h, in parallel with passive sampling to allow validation and quantification of EDC measurements.

### 4.4 Physicochemical markers

Low-cost indoor air quality sensors were used for continuous monitoring at one-minute intervals of PM₁₀, PM₄, PM₂.₅, PM₁, CO₂, air temperature, relative humidity, and volatile organic compounds (VOCs). Each sensor integrated a Sensirion SPS30 particulate matter sensor for both mass and number concentrations (69), a MH-Z19-C non-dispersive infrared (NDIR) CO₂ sensor (70), either an AM2320 or ENS210 air temperature/humidity sensor (71, 72), and a Renesas ZMOD4410 sensor for total VOC (TVOC) and ethanol-equivalent concentrations (73). The MH-Z19-C sensor applies an automatic baseline calibration (ABC) algorithm, which stabilizes CO₂ measurements within approximately 1 week after installation by periodically adjusting the sensor baseline based on minimum nighttime concentrations.

Upon receipt from the supplier and prior to deployment, all sensors were checked and compared to research grade instruments. All sensor units were tested side-by-side in batches of approximately 30 sensors under controlled indoor conditions for ~4.5 h and compared against a Grimm 11-D particle sensor (74) for particulate matter measurements and a Vaisala HMP1 (75) for CO₂, air temperature, and relative humidity measurements. Sensors showing unstable signals, substantial deviation from reference measurements, communication issues, or other malfunctioning behavior were considered unreliable and excluded from the study.

In 165 out of 180 classrooms, two sensor units were installed per classroom on opposite sides of the room to capture potential spatial variability and ensure redundancy in case of device failure or disconnection. Sensors were deployed continuously during the 9-week intervention period and for an additional 2-week pre- or post-intervention period, resulting in a total monitoring duration of 11 weeks per classroom. Classroom ventilation was estimated with the average air change rate (ACH) calculated from CO₂ decay curves, applying the standard exponential decay method following source removal (76). A running window was applied to identify CO_2_-decay curves during the monitoring period from which ACH could be derived.

### 4.5 Environmental and health monitoring

Contextual data will be considered to aid interpretation of observed airborne microbial and PM measurements, including weekly national infectious-disease surveillance reports (77–79), as well as ambient PM and NO₂ data obtained from national monitoring networks, with outdoor NO₂ used as an indicator of traffic-related pollution that may influence indoor PM levels (80). Classroom-level absenteeism (anonymized) was recorded. Parent-reported respiratory symptoms, including wheezing, asthma, allergic complaints, and respiratory infections, were collected via retrospectively administered surveys at the end of the 2024–2025 winter season. Two surveys were administered, covering respiratory symptoms during the winters of 2023–2024 and 2024–2025, respectively. Depending on the school, the 2023–2024 winter represented either a pre-intervention or an intervention period. The questionnaire was adapted from a previous survey (81), which included selected items from the International Study of Asthma and Allergies in Childhood (ISAAC) questionnaire (82). Survey data were pseudonymized prior to analysis. Detailed descriptions of the survey and epidemiological analysis will be reported in future work.

## 5 Power calculation and sample size justification

Simulations were used to estimate the sample size (i.e., number of schools, classrooms and intervention periods) prior to the study. A power of 80% was targeted to detect a minimal average (proportional) reduction of 20% in microbial marker levels attributable to the intervention, under the null hypothesis that the intervention has no effect across a range of different scenarios. These scenarios were formulated to include a range of possible values for the expected between-school, between-classroom, and within-classroom variances to account for the hierarchical (clustered) design, and to evaluate a limited set of design options. Information regarding the possible values for variance parameters were obtained from earlier studies by Jacobs et al. (4) on endotoxin concentrations in Dutch primary classrooms and by Linde et al. (unpublished data) on 16S rRNA data in Dutch secondary schools. Design options included the number of clusters of classrooms to enroll per school (varying from 1 to 3), the number of repeated measurements during intervention per classroom (either 3 or 4), and whether an (additional) baseline measurement before intervention was available or not. All simulations were based on a design where the number of intervention and control classrooms were the same for each school. Power was defined as the proportion of simulations in which a t-test for the intervention effect in a suitably formulated mixed effect model, and using the Satterthwaite approximation, resulted in a *p*-value < 0.05. Starting from an initial sample size of 16, the sample size needed to achieve 80% power was then estimated using an iterative algorithm and using 1,000 simulations per run. All simulations were performed in R (version 4.3.1) and using the lme4 and lmerTest packages.

The simulations, which considered different assumptions about variance and the number of classrooms per school, indicated that achieving 80% power could require between 6 and 25 schools, depending on whether schools contributed 1, 2, or 3 classroom clusters (see Supplementary Table S4; Supplementary Figures S1, S2 for details). Results indicated that including two clusters per school rather than one substantially increased power, whereas adding a third cluster per school had a smaller additional effect. Increasing the number of repeated measurements per classroom from three to four also increased power modestly. Including an additional baseline measurement prior to intervention further increased power. Based on these simulations the study design included a baseline measurement plus three repeated intervention measurements per classroom.

## 6 Data analysis plan

All analyses will be performed in RStudio [version 4.5.1 (83)]. The project will use the UU network drive for secure data archiving, with access restricted to authorized study personnel. Data were coded using consistent study identifiers prior to analysis, with a separate pseudonymization key linking identifiers to study sites.

Intervention effects taking into account data of all participating schools will be assessed using generalized linear mixed-effects models (GLMMs) with random intercepts for school, cluster, and classroom, reflecting the hierarchical clustering inherent to the study design. Residual normality and homoscedasticity will be verified, applying log transformation when necessary to meet model assumptions. To account for temporal correlation between successive intervention periods within classrooms, autoregressive structures [e.g., AR (1)] will be considered, with their inclusion guided by both model fit and the anticipated influence of seasonal variation. Potential covariates, including grade, classroom dimensions, number of students, student density, lesson hours, floor level, urbanization, ventilation type, and flooring type will be evaluated in univariate GLMMs containing intervention arms and random effects. Covariates significantly impacting intervention estimates or model fit may be considered for inclusion in the final multivariable model, guided by both statistical evidence and conceptual relevance. ACH will be considered as a potential covariate in the mixed-effects models to account for differences in baseline ventilation and may be evaluated as a continuous variable or categorized into predefined ranges (ACH-bins). Multicollinearity will be assessed, with highly correlated variables excluded to ensure model stability, and the final model will be selected based on overall fit (AIC, likelihood ratio tests).

Sensitivity analyses will assess model robustness by re-fitting the final models after exclusion of outliers (identified by influence diagnostics), exclusion of baseline measurements, and restriction to the 2024–2025 sampling period only. An additional sensitivity analysis will be performed to assess the robustness of the findings under protocol-compliant PAC operation. Only measurements without indication of incorrect PAC operation are included, based on the recorded device settings during sample placement and collection by the research team and the absence of reported deviations in the teacher log forms.

Data analysis of species-specific bacterial and viral markers will be guided by observed detection levels. Where quantitative measurements permit, marker concentrations will be analyzed using GLMMs as described above. If detection within the quantifiable range is limited, outcomes will be modeled as binary (detected or non-detected) using logistic mixed-effects models with random effects for school, cluster, and classroom, and fixed effects for the intervention arms. Appropriate model assumptions and hierarchical variance structures will be considered. Correlations between markers may be examined to explore co-detection patterns. Missing data will not be imputed; analyses will use available data within mixed-effects models.

In addition to standard GLMM or binary logistic mixed-effects models, Bayesian hierarchical models may be applied to species-specific outcomes as continuous variables, allowing incorporation of observations outside the quantifiable range while still leveraging the information from measurable values. Observations within the quantifiable range will be treated as measured values, whereas non-detects or values outside of the quantifiable range, will be incorporated as censored data, reflecting the uncertainty in the true underlying value. Feasibility of using a Bayesian approach in this case depends on sufficient informative observations within the measurable range. Random intercepts for school, cluster and classroom will account for the hierarchical structure. Priors for intercepts, intervention effects, residual variance, and group-level variance will be informed by plausible ranges derived from the quantifiable data. Model fit will be evaluated via posterior predictive checks, and sensitivity analyses may explore the impact of prior choices and censoring assumptions.

## 7 Discussion

This cRCT study design intends to evaluate PAC effectiveness in primary school classrooms under real-world conditions while preserving methodological rigor. Compared with laboratory-based studies, this field-based approach strengthens validity by reflecting real classroom dynamics, including occupant behavior, ventilation variability and practical use of PACs. The study will employ a dual-control design (Figure 1), that will combine measurements in all classrooms during a non-intervention period as well as control classrooms throughout the study. This design enables differentiation between pre-existing classroom differences and temporal trends. Although not blinding students and school staff to PAC placement may have introduced some behavioral changes. Repeated measurements enhance statistical power and temporal resolution. Clustering classrooms by key characteristics improves comparability between intervention and control groups. Random allocation of intervention arms minimizes allocation bias, although perfect comparability between classrooms within a cluster is unattainable.

Importantly, this design also addresses limitations inherent to several commonly applied in-situ study designs, in which natural variability in particulate and microbial concentrations may be incorrectly attributed to PAC operation. Between-group comparisons are sensitive to pre-existing classroom- or school-level differences. Pre–post designs are susceptible to temporal confounding. Although classroom-level crossover designs effectively control for classroom characteristics, they are limited in their ability to capture temporal variability as well. Airborne PM concentrations in the Netherlands exhibit substantial temporal variability driven by meteorological factors such as wind direction and air temperature, with PM₁₀ concentrations during short-term episodes sometimes increasing several-fold relative to the annual average over periods of several days (84). Short-term temporal variation in PM₂.₅ is detectable at the scale of several days, and these multi-day fluctuations in ambient PM₂.₅ are consistently correlated with personal exposure (85). Airborne bacterial concentrations indoors can also exhibit pronounced short-term temporal variability, even within the same season. Emerson et al. (86) demonstrated that bacterial abundances varied substantially within individual homes over time, with samples collected only weeks apart often being similarly different as samples collected months apart. These findings underscore the importance of repeated sampling in classroom studies to account for natural temporal variability, which our design integrates to avoid over- or underestimation of PAC effectiveness.

Nonetheless, airborne microbial or viral spillover between classrooms cannot be entirely excluded in our cRCT design with randomization at the classroom level and remains unquantified. Simulation-based multizone modeling studies highlighted that while natural ventilation may reduce airborne pathogen concentrations within classrooms, airflow through corridors and shared spaces may also facilitate airborne transport between classrooms, potentially mediating cross-classroom infection risk (87). In the present study, priority was given to comparability between intervention and control classrooms with respect to contextual conditions and to account for temporal variability in airborne markers, rendering classroom-level randomization the most appropriate design choice. For airborne exposure outcomes, PAC-driven reduction remains estimable, even in the presence of inter-classroom spillover, as classrooms equipped with PACs are expected to exhibit lower airborne concentrations than controls. By contrast, studies primarily targeting infection incidence and student absenteeism may warrant school- level randomization to minimize dilution from inter-classroom spillover. Variance decomposition analyses from prior school-based studies further support this approach: while relative contribution of school-, classroom-, and within-classroom variance differs by marker, both endotoxin and 16S data show that within-classroom (day-to-day) variability constitutes a highly substantial proportion of total variance (4), suggesting that time-varying and other unmodelled processes are major contributors to airborne concentration dynamics and supporting classroom-level randomization with repeated measurements.

The distribution of ventilation types in participating schools (3.7% balanced mechanical, 11.1% mechanical exhaust only, 74.1% mechanical (type unknown), 11.1% natural) suggests a higher prevalence of mechanically ventilated classrooms compared with the national snapshot reported by Krishnan et al. (88), who found 40% natural, 16% fully mechanical systems, 4% mechanical supply only and 40% mechanical exhaust only across 25 Dutch school buildings. However, self-reported ventilation types may not reliably reflect actual performance, as schools may not be fully aware of system operational status and maintenance. To evaluate the ventilation performance in classrooms, ACH will be determined from the CO₂ decay curves. As ventilation conditions may influence PAC effects, with greater effects generally observed at lower ventilation rates (89, 90), the planned analyses may account for differences in baseline ventilation by adjusting for ACH.

To monitor airborne microbial exposure, EDCs were employed. EDCs enable low-cost, silent, and electricity-free sampling, facilitating standardized deployment on a large scale over extended periods without disrupting teaching activities. While EDCs provide integrated rather than real-time measurements, they are well-established for monitoring airborne microbial material, such as endotoxins (67, 91–93), bacterial (94–97) and viral (98) particles, with moderate to strong correlations to active samplers (r ≈ 0.54–0.84) in household and farm environments (67, 93, 95). Viral detection with passive samplers may remain challenging due to low viral prevalence and inherent variability in classrooms (99), whereas bacterial and 16S markers are more consistently measurable and provide a proxy for airborne microbial exposure (94–97). While spatial heterogeneity within classrooms, influenced by non-homogeneous air mixing, has been observed empirically and supported by both standard and AI-trained simulations (100–102), sampling was designed to capture representative locations, allowing estimation of average classroom exposure and meaningful comparisons between intervention and control classrooms. Preliminary testing indicated that, while occasional deviations in EDC placement occurred due to practical constraints (e.g., ceiling height), placement was generally consistent within clusters. Overall, EDCs offer a feasible, reproducible, and minimally disruptive approach for large-scale classroom airborne microbial monitoring.

This study’s rigorous pre-selection, laboratory testing, and standardized operating procedures allowed comparable performance across classrooms. Although practical constraints on PAC placement may limit optimal device efficiency, they reflect realistic real-world implementation conditions, with effect sizes likely distributed across a range of classroom settings. Importantly, any observed PAC-driven reduction in airborne exposure must be interpreted in the context of practical considerations, including device size, noise, electricity use, maintenance requirements, and long-term costs, which shape whether and how PACs are adopted and maintained in schools. For example, regular maintenance is essential to sustain PAC performance, as air cleaning performance and CADR may gradually decline over prolonged operation as particulate loading accumulates (103). In addition, successful implementation depends on user acceptance. Device noise, for example, may reduce user acceptance, discourage consistent PAC operation, and contribute to perceived classroom disruption. Previous studies indicate that noise levels up to ~40 dB generally cause minimal disturbance, whereas higher flow rates can result in noticeable disruption through noise or draught-related discomfort (51, 101).

By combining PM, microbial markers, and contextual data, this trial provides a comprehensive assessment of both exposure and potential health impacts, including absenteeism and symptom reporting. While microbial and PM measurements serve as objective indicators of exposure, translating this into reduced infection risk remains complex due to uncertainties in dose–response relationships, pathogen viability, and personal susceptibility. Nonetheless, repeated, multi-classroom measurements in this study may inform and refine predictive models of airborne transmission in classrooms. Our trial generates essential data to support cost–benefit analyses and evidence-informed decision-making, helping to identify contexts where PACs are most warranted, such as schools with suboptimal air quality or during periods of heightened respiratory infection risk. PACs are always intended as a complement to adequate ventilation, which remains essential for CO₂ control. Ultimately, these insights aim to guide strategic implementation to maximize public health benefits.

## 8 Ethics and dissemination

Ethical approval was obtained from the Ethics Review Board of Eindhoven University of Technology (TU/e; project #10031209). The Medical Research Ethics Committee NedMec (MREC NedMec, The Netherlands) confirmed that the study did not fall under the scope of the Dutch Medical Research Involving Human Subjects Act (WMO; reference 23-228/DB). The study was retrospectively registered at ClinicalTrials.gov (NCT07479420; date of registration: 6 March 2026). Institutional informed consent was obtained from all participating schools by the research team prior to inclusion in the study.

The study was conducted within a multi-institutional research consortium under the oversight of a steering committee comprising representatives from each participating institution. As the study involved no meaningful participant safety risks, no separate data monitoring committee was established. Members of the public were not involved in the design or conduct of the research, as this was not considered feasible. Study continuity relied on routine scheduling of sampling visits with schools, with no additional retention strategies implemented.

School-level metadata and environmental measurement data are stored securely at institutional research data storage locations. Study results will be reported in de-identified format. Access to identifiable data is restricted to a small number of authorized members of the research team involved in field work. Results will be disseminated through peer-reviewed publications and scientific conferences. A lay summary of study findings will be shared with participating schools. Results will be presented and discussed with the private partners involved in the project.

## 9 Conclusion

This study presents a rigorous design to evaluate PAC effectiveness on-site in primary school classrooms. By combining repeated, clustered measurements with both baseline and control comparisons, it supports reliable estimation of intervention effects under realistic conditions. This approach provides a research framework for the evaluation of PACs as a complementary measure to ventilation in schools and contributes to improving indoor air quality research in educational settings.
