# Supplementary material for: Evaluating air cleaner effectiveness in schools: a comprehensive review and protocol of a cluster-randomized controlled trial of physicochemical and microbial markers
Source: Front Public Health. 2026 Aug 25;14:1892902. doi: 10.3389/fpubh.2026.1892902 (PMC13550210; doi:10.3389/fpubh.2026.1892902)
Supplement: Supplementary file 1 [file Data_Sheet_1.PDF]

SUPPLEMENTARY METHODS

Literature survey

To identify studies on PACs in schools, a structured but non-systematic literature search was conducted. The main databases searched were Google Scholar and PubMed. This search strategy was intended to provide an illustrative overview of the current state of knowledge from field studies reporting PAC effects on indoor environmental exposures in schools. Additional studies were identified from references in included articles and through recommendations from experts in the field, and by consulting AI-assisted literature suggestions, which were then manually screened for relevance.

Table S1. Summary of search strategy and inclusion/exclusion criteria

Component	Description
Databases	Google Scholar, PubMed
Search terms	"air purifier" school (first 10 pages of Google Scholar screened)<br>“air cleaner” classrooms (first 3 pages of Google Scholar screened)<br>"air cleaner" school bioaerosol (all results on PubMed screened)<br>"air cleaner" school virus (all results on PubMed screened)<br>"air cleaner" school bacteria (all results on PubMed screened; 0 relevant articles)
Inclusion criteria	1. Primary, middle, or secondary schools<br>2. Portable air cleaner (PAC) (non- integrated into mechanical ventilation systems)<br>3. PAC effect distinguishable from other interventions<br>4. Exposure: PM, microbial agents (bacteria, viruses), or aerosol decay metrics
Exclusion criteria	1. Non-peer-reviewed: conference abstracts, theses, reports<br>2. Non-school settings<br>3. PAC effect not separable from other interventions<br>4. Mixed settings (school + non-school) without separated results<br>5. Health outcomes only (non-environmental)<br>6. Focus on other pollutants (e.g. UFP only)

Table S2. Reagents and kits used for nucleic acid extraction

Reagent / kit	Supplier	Catalogue number	Specification
DPBS (1×)	Gibco (Fisher Scientific)	14190144	Without Ca^{2+}/Mg^{2+}, phenol red
Lysis buffer BL	LGC Genomics GmbH	NAP10-002-04	Part of LGC extraction protocol
Quick-RNA MiniPrep Kit	Zymo Research (Sanbio)	R1055	With DNase treatment
LGC DNA kit buffers	LGC Genomics GmbH	—	Buffers ordered separately

Table S3. qPCR and RT-qPCR assay conditions per microbial target

Target	Assay type	Chemistry	RT step	Cycling conditions	Fluorophore	Melt curve	Cycles
Influenza A	RT-qPCR	Fast One-Step	5 min 50 °C	95 °C 20 s; 50× (95 °C 3 s, 60 °C 30 s)	FAM	No	50
Influenza B	RT-qPCR	Fast One-Step	5 min 50 °C	95 °C 20 s; 50× (95 °C 3 s, 60 °C 30 s)	FAM	No	50
RSV	RT-qPCR	Fast One-Step	5 min 50 °C	95 °C 20 s; 50× (95 °C 3 s, 60 °C 30 s)	Texas Red	No	50
16S rRNA	qPCR	SYBR Green	—	95 °C 3 min; 50× (95 °C 15 s, 72 °C 30 s)	SYBR	Yes	50
S. *aureus*	qPCR	TaqMan	—	95 °C 10 min; 50× (95 °C 15 s, 60 °C 1 min)	Cy5	No	50
S. *epidermidis*	qPCR	TaqMan	—	95 °C 10 min; 50× (95 °C 15 s, 60 °C 1 min)	Cy5	No	50
S. *salivariu*s	qPCR	TaqMan	—	95 °C 10 min; 50× (95 °C 15 s, 60 °C 1 min)	Cy5	No	50
M. *catarrhalis*	qPCR	TaqMan	—	95 °C 10 min; 40× (95 °C 15 s, 60 °C 1 min)	FAM	No	50

Supplementary Figures S1–S2 provide a visual summary of simulation-based power analyses, followed by Supplementary Table S4 detailing the underlying variance assumptions and design parameters with a baseline measurement included.

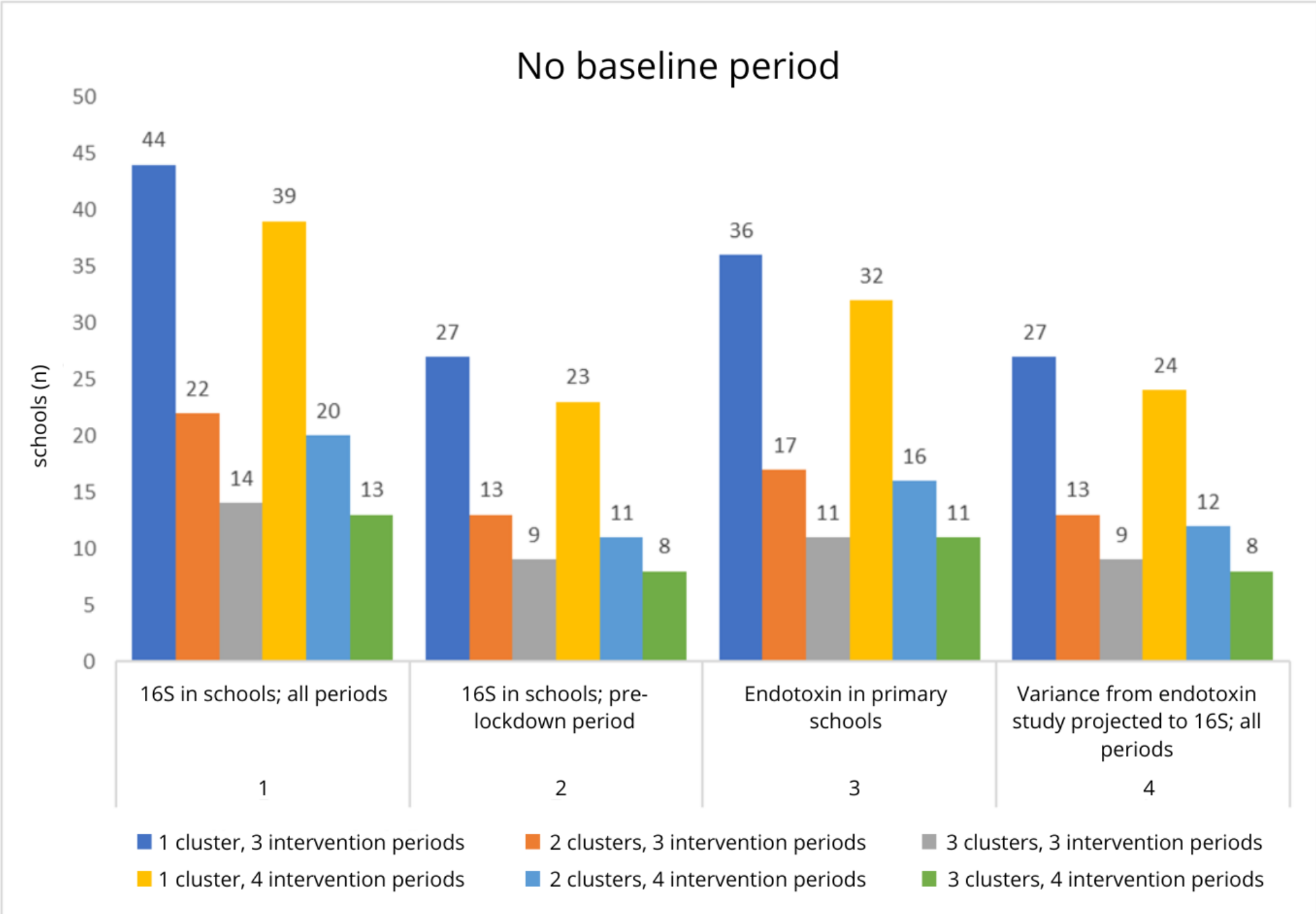

Figure S1. Visual summary of power simulations without baseline. *Simulation-based estimates of the number of schools required to achieve approximately 80% statistical power under different variance scenarios and study design options without a baseline measurement***.** *Power calculations assume a 20% intervention effect and vary the number of classroom clusters per school and the number of repeated intervention measurements. Variance scenarios are described in Supplementary Table S4.*

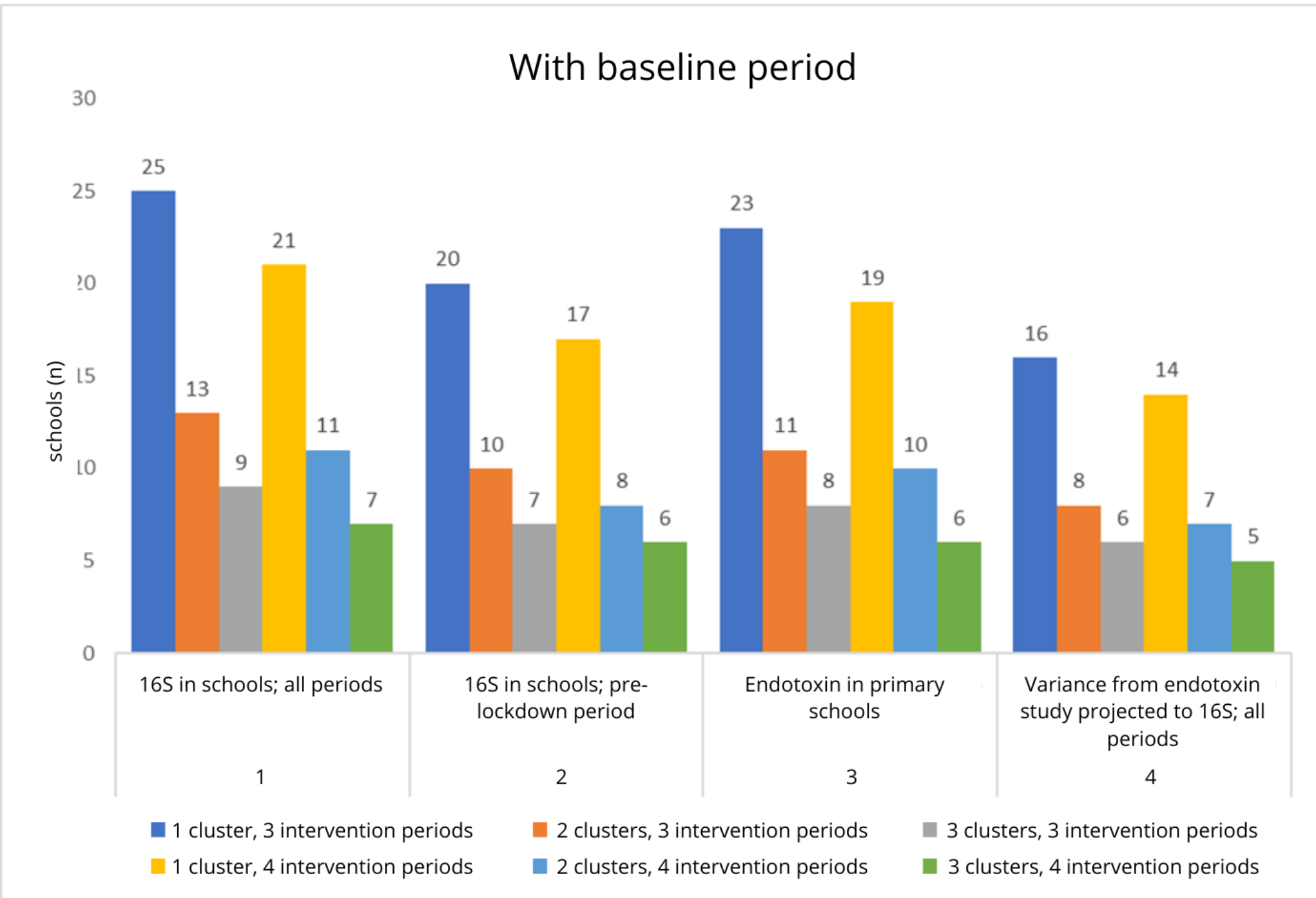

Figure S2. Visual summary of power simulations with baseline. *Simulation-based estimates of the number of schools required to achieve approximately 80% statistical power under different variance scenarios and study design options including a baseline measurement***.** *Power calculations assume a 20% intervention effect and vary the number of classroom clusters per school and the number of repeated intervention measurements. Variance scenarios are described in Supplementary Table S4.*

Table S4. Power simulation scenarios including baseline measurement

Scenario	Schools (n)	Classroom clusters	Repeated measurements (excl. baseline)	Power
1	25	1	3	0.797
2	20	1	3	0.809
3	23	1	3	0.798
4	16	1	3	0.801
1	13	2	3	0.801
2	10	2	3	0.803
3	11	2	3	0.794
4	8	2	3	0.780
1	9	3	3	0.811
2	7	3	3	0.820
3	8	3	3	0.811
4	6	3	3	0.833
1	21	1	4	0.795
2	17	1	4	0.800
3	19	1	4	0.797
4	14	1	4	0.800
1	11	2	4	0.802
2	8	2	4	0.781
3	10	2	4	0.813
4	7	2	4	0.794
1	7	3	4	0.784
2	6	3	4	0.830
3	6	3	4	0.771
4	5	3	4	0.817

1. Scenarios are based on four datasets, reflecting different assumptions for variance components from previous studies:
    1) Scenario 1: 16S in schools, all periods; between-school variance = 0.0036, between-class variance = 0.0169, within-class variance = 0.0256, total variance = 0.0461 (Linde et al., unpublished data)
    2) Scenario 2: 16S in schools, pre-lockdown period; between-school variance = 0.0049, between-class variance = 0.0064, within-class variance = 0.0256, total variance = 0.0369 (Linde et al., unpublished data)
    3) Scenario 3: Endotoxin in primary schools; between-school variance = 0.0270, between-class variance = 0.0120, within-class variance = 0.0240, total variance = 0.0630 (4)
    4) Scenario 4: Variance from endotoxin study projected to 16S all periods; between-school variance = 0.0198, between-class variance = 0.0088, within-class variance = 0.0176, total variance = 0.0461 (Linde et al., unpublished data; (4))
2. All scenarios include a baseline measurement plus three repeated intervention measurements per classroom.
3. Power was calculated as the proportion of 1,000 simulated datasets in which the intervention effect was detected at $p < 0.05$ using a mixed-effects model with Satterthwaite degrees of freedom.
4. AR(1) autocorrelation was tested but had negligible effect and is omitted.
5. Minimal detectable difference set at 20% reduction for all scenarios.
